# Cytokine Responses to Novel Antigens in an Indian Population Living in an Area Endemic for Visceral Leishmaniasis

**DOI:** 10.1371/journal.pntd.0001874

**Published:** 2012-10-25

**Authors:** Om Prakash Singh, Carmel B. Stober, Abhishek Kr. Singh, Jenefer M. Blackwell, Shyam Sundar

**Affiliations:** 1 Infectious Disease Research Laboratory, Department of Medicine, Institute of Medical Sciences, Banaras Hindu University, Varanasi, India; 2 Department of Medicine, University of Cambridge School of Clinical Medicine, Cambridge, United Kingdom; 3 Cambridge Institute for Medical Research, University of Cambridge School of Clinical Medicine, Addenbrooke's Hospital, Cambridge, United Kingdom; 4 Telethon Institute for Child Health Research, Centre for Child Health Research, The University of Western Australia, Subiaco, Western Australia, Australia; Hospital Universitário, Brazil

## Abstract

**Background:**

There are no effective vaccines for visceral leishmaniasis (VL), a neglected parasitic disease second only to malaria in global mortality. We previously identified 14 protective candidates in a screen of 100 *Leishmania* antigens as DNA vaccines in mice. Here we employ whole blood assays to evaluate human cytokine responses to 11 of these antigens, in comparison to known defined and crude antigen preparations.

**Methods:**

Whole blood assays were employed to measure IFN-γ, TNF-α and IL-10 responses to peptide pools of the novel antigens R71, Q51, L37, N52, L302.06, J89, M18, J41, M22, M63, M57, as well as to recombinant proteins of tryparedoxin peroxidase (TRYP), *Leishmania* homolog of the receptor for activated C kinase (LACK) and to crude soluble *Leishmania* antigen (SLA), in Indian patients with active (n = 8) or cured (n = 16) VL, and in modified Quantiferon positive (EHC^+ve^, n = 20) or modified Quantiferon negative (EHC^−ve^, n = 9) endemic healthy controls (EHC).

**Results:**

Active VL, cured VL and EHC^+ve^ groups showed elevated SLA-specific IFN-γ, but only active VL patients produced IL-10 and EHC^+ve^ did not make TNF-α. IFN-γ to IL-10 and TNF-α to IL-10 ratios in response to TRYP and LACK antigens were higher in cured VL and EHC^+ve^ exposed individuals compared to active VL. Five of the eleven novel candidates (R71, L37, N52, J41, and M22) elicited IFN-γ and TNF-α, but not IL-10, responses in cured VL (55–87.5% responders) and EHC^+ve^ (40–65% responders) subjects.

**Conclusions:**

Our results are consistent with an important balance between pro-inflammatory IFNγ and TNFγ cytokine responses and anti-inflammatory IL-10 in determining outcome of VL in India, as highlighted by response to both crude and defined protein antigens. Importantly, cured VL patients and endemic Quantiferon positive individuals recognise 5 novel vaccine candidate antigens, confirming our recent data for *L. chagasi* in Brazil, and their potential as cross-species vaccine candidates.

## Author Summary

Visceral leishmaniasis is a parasitic infection that results in death in susceptible people unless they are treated. Current drugs are expensive and toxic, and there are no vaccines in use in humans. We know that it is possible to become immune to infection with this parasite because people who have been cured using drug treatment are resistant to further infection. In addition, a large percentage of people infected with the parasite remain asymptomatic and develop a specific immune response that can be measured using crude leishmanial antigens. We hypothesized that these resistant people might hold the key to understanding the kind of immune response required for protection. In this paper we compared the immune response to a series of novel vaccine candidates in people with active disease, in those drug-cured from the disease, and in the naturally resistant individuals. We show that immune individuals make strong cytokine responses to five of eleven novel vaccine candidates that were tested, making them ideal candidates to take forward in the development of a defined vaccine against leishmaniasis.

## Introduction

Visceral leishmaniasis (VL), also known as kala-azar, is a potentially fatal disease caused by obligate intracellular parasites of the *Leishmania donovani* species complex. VL is a serious public health problem in indigenous and rural populations in India, accounting for enormous morbidity and mortality, as well as major costs to both local and national health budgets. The estimated annual global incidence of VL is 200,000 to 400,000, and >90% of these cases occur in India, Bangladesh, Sudan, South Sudan, Ethiopia and Brazil [1]. Interestingly, 80 to 90% of human infections are subclinical or asymptomatic, and this asymptomatic infection is associated with strong cell-mediated immunity [2], [3], [4], [5], [6]. Only a small percentage of infected individuals develop severe disease [7], [8], and patients who recover from VL display resistance to reinfection [5]. This suggests the development of protective immunity and provides a rational basis for the development of vaccines that impart potent cell-mediated immune responses. Furthermore, the factors that skew the immune response toward T helper 1 (Th_1_) or Th_2_/T regulatory (T_reg_) cell dominance are partially understood, and it is believed that direct interaction between parasite antigens and host immune cells participate to shape the subsequent pathogenic or protective immune responses [9], [10].

A key mechanism by which T cells mediate their effector functions is through the production of cytokines. However, heterogeneity of CD4+ T-cell cytokine responses has made it difficult to define immune correlates of protection after vaccination in leishmaniasis. In murine cutaneous leishmaniasis, the degree of protection in vaccinated mice was predicted by the frequency of CD4+ T cells simultaneously producing interferon-γ (IFN-γ), interleukin (IL)-2 and tumour necrosis factor (TNF, formerly TNF-α) [11]. These multi-functional effector CD4+ T cells elicited by all vaccines tested were unique in producing high amounts of IFN-γ [11]. In our own studies comparing vaccines with different efficacies in mice, we found that the balance between antigen-specific CD4 T cell-derived pro-inflammatory IFN-γ and regulatory IL-10 (and to a lesser extent IL-4 and IL-5), rather than magnitude of IFN-γ *per se*, provided the best correlate of a protective immune response [12]. A strong tumour necrosis factor-α (TNF-α) response concurrent with IFN-γ has also been shown to be important in models of VL [13]. A crucial step in vaccine development against human disease requires improved understanding of the functional heterogeneity of T-cell cytokine responses generated by candidate vaccine antigens. For example, one study in malaria reported that peptide-specific IFN-γ to a conserved epitope of the circumsporozoite surface protein was strongly associated with protection of humans again infection and disease [14], providing a precise target for vaccine design. Without a convincing single marker of protective immunity against leishmaniasis, vaccine development has to rely on screening a range of cytokines to gauge the balance between Th_1_ and Th_2_/T regulatory (T_reg_) responses.

Advances in our understanding of *Leishmania* pathogenesis and of the generation of host protective immunity, together with completed *Leishmania* genome sequences, have opened new avenues for vaccine research. Although significant progress has been made to understand mechanisms of VL immunity in humans [9], [10], there is no effective vaccine available for humans against any form of leishmaniasis. Drugs used in leishmaniasis therapy are significantly toxic, expensive and faced with increasing resistance. Limitations in pharmacotherapy argue for the development of a vaccine for VL. Vaccination with live virulent parasites, termed leishmanization, was practiced from ancient times until recently in many endemic areas [15]. Vaccine trials involving whole, killed parasites were conducted in the 1970s and 1980s [16], [17]. Although no overall statistically significant protection has been associated with any trial of these killed vaccines [18], [19], [20], [21], [22], a common theme has been protection in persons who showed conversion of Leishmania-specific delayed type hypersensitivity (DTH) skin test responses during the trial, whether or not they received the vaccine. The latter points to the importance of understanding the immune response in exposed individuals who become infected but do not progress to clinical disease, which in the Indian endemic area has been equated to a positive modified Quantiferon response to leishmanial antigens in whole blood assays [23].

The genome sequence and proteome data (∼33.6 Mb genome and ∼8300 protein coding genes) of *Leishmania major* [24] provides a rich source of potential vaccine candidates. We recently described the identification of novel *Leishmania* antigens delivered as DNA vaccines to susceptible BALB/c mice, and identified 14 protective candidate antigens in a screen of 100 amastigote-expressed genes [25]. To determine their potential as vaccine candidates for humans, we here evaluate the ability of 11 of these novel *Leishmania* vaccine candidates, along with soluble *Leishmania* antigen (SLA), recombinant *Leishmania* homolog of the receptor for activated C kinase (LACK), and tryparedoxin peroxidase (TRYP) proteins, to stimulate cytokine responses in whole blood from active and cured VL patients, and from modified Quantiferon positive and negative endemic health controls (EHC), in India.

## Materials and Methods

### Study subjects

The study was approved by the Ethics Committee of the Banaras Hindu University, Varanasi, India. Written informed consent was obtained from all adult subjects included in the study, or from the parents or guardians of individuals less than 18 years of age. Subjects belonged to 4 clinically well characterized groups: (i) active VL: cases of parasitologically confirmed, active VL (n = 8); (ii) cured VL: subjects who were definitively cured of VL and shown to have no parasites in splenic aspirates at least 6 months after treatment (n = 16); (iii) EHC with a positive antigen-specific IFN-γ response measured by modified Quantiferon (Cellestis, Chadstone, Australia) assay (cf. below) (EHC^+ve^, n = 20); and (iv) EHC testing negative by modified Quantiferon assay (EHC^−ve^, n = 9). Subjects having fever within the past month, and children less than five years of age, were excluded. Follow up visits were made to the homes of the EHC^+ve^ and cured subjects 6 and 12 months after enrolment to monitor for the development of active VL. Demographic and clinical characteristics of participants enrolled in the vaccine study are summarized in Table 1. None of the cured VL or EHC subjects developed clinical VL during the 1 year follow-up.

**Table 1:** Demographic and clinical characteristics of participants.

	Post treatment (Cured VL)	Active VL	ENDEMIC HEALTHY CONTROLS	
			Quantiferon +ve (EHC^+ve^)	Quantiferon −ve (EHC^−ve^)
**N**	**16**	**08**	**20**	**09**
**Age (year)**	23.81±10.57	21.13±7.93	26.96±11.74	24.56±11.73
**Sex % (M/F)**	45∶55	25∶75	37∶63	34∶56
**Weight (Kg)**	41.94±12.77	43.00±10.73	40.21±8.135	44.67±11.42
**RBC** * **(×10^6^/mm^3^)**	4.29±0.537	3.06±0.339	4.22±0.581	4.03±0.79
**Platelets (×10^5^/mm^3^)**	2.50±0.85	1.18±0.77	2.3±1.17	2.6±1.07
**WBC** * **(×10^3^/mm^3^)**	10.33±2.68	3.8±1.84	9.97±2.56	11.56±4.37
**Haemoglobin (g/dl)**	11.69±1.25	7.87±1.752	11.79±1.229	11.53±1.9
**Lymphocyte (×10^3^/mm^3^)**	2.69±0.72	1.70±0.63	2.86±0.91	2.76±0.51
**Granulocyte(×10^3^/mm^3^)**	6.95±2.76	1.76±1.33	6.32±2.23	7.8±3.8
**SGOT** * **(IU/ml)**	ND	53.0±43.79	ND	ND
**SGPT** * **(IU/ml)**	ND	48.13±44.12	ND	ND
**Creatinine (mg/dl)**	ND	0.85±0.23	ND	ND
**Splenic Score** #	NA	2.75±1.28	NA	NA

* RBC = Red Blood Cells; WBC = White Blood Cells; SGOT = Serum glutamic oxaloacetic transaminase; SGPT = Serum glutamic pyruvate transaminase.

# Splenic score was graded on a conventional logarithmic scale of 0 (indicating no parasites per 1000 oil-immersion fields) to 6 (indicating >100 amastigotes per 1000 fields) at ×1000 magnification.

**Note:** Mean value ± SD of aggregated data are shown throughout, N/D = not done, N/A = not applicable.

### Preparation of soluble leishmania antigen (SLA)

SLA from an Ethiopian strain of *L. donovani* (LV9) or *L. major* (LV39) were prepared at the Cambridge Institute for Medical Research, University of Cambridge School of Clinical Medicine, UK as described previously [12]. SLA from an Indian strain of *L. donovani* was prepared at the Infectious Disease Research Laboratory, Banaras Hindu University, according to the published protocol of Scott and co-workers [26]. The protein concentration was estimated using the BCA method [27]. SLA was stored at −80°C until use.

### Vaccine antigens

Recombinant LACK and TRYP proteins were prepared as described [11], [12], with large-scale preparation, endotoxin removal and protein estimation out-sourced to Novexin Ltd. (Cambridge, UK). As previously described [28], overlapping 13–20-mer peptides (minimal overlap of 12 amino acids to ensure complete coverage of epitopes, 7–31 peptides/antigen depending on amino acid length of the protein) representing 11 (R71, Q51, L37, N52, L302.06, J89, M18, J41, M22, M63, M57) of 14 novel antigens identified [25] were synthesized commercially (Peptide2.0, Chantilly, VA), initially solubilized in dimethylsulfoxide (final concentration in the well <0.1% DMSO), and pooled (for peptides within each antigen) in endotoxin-free phosphate-buffered saline at a final concentration of 50 µg/mL per individual peptide. Peptides pools were stored at −80°C. Due to the size of peptides in the pools, some natural processing is required for epitope selection prior to epitope binding to class II and presentation to CD4+ T cells.

### Diluted whole blood assay

The Quantiferon (Cellestis, Chadstone, Australia) whole blood assay was conducted on 147 endemic healthy individuals according to the manufacturer's instructions or to our published modifications [6], [23]. From these data, 20 highly positive (above the cut-off value generated by the ROC curve) individuals were selected as the EHC^+ve^ study group, and 9 individuals selected at random from below the cut-off value as the EHC^−ve^ study group. Blood (5 mL) was collected into heparinised tubes, and samples diluted 1 in 8 in serum-free complete medium comprising RPMI supplemented with 2 mM L-glutamine, 100 µg/mL streptomycin, 100 IU/mL penicillin (Gibco, USA). Diluted blood (180 µL/well) was plated into 96-well U-bottomed plates (Nunc, Rochester, USA) and antigen added in triplicate wells at a final concentration of 10 µg/mL for TRYP, LACK and SLA, 5 µg/mL for PPD, PHA and the 11 novel antigen peptide pools, and made up to a volume of 200 µL. Plates were incubated at 37°C in 5% CO2 for 24 hours, 72 hours or 6 days. Supernatants from replicate wells were harvested, pooled and stored at −80°C until analysed by ELISA.

### Measurement of cytokines by ELISA

Cytokine release (IFN-γ, TNF-α and IL-10) by antigen stimulated whole blood cells was measured at 24 hours, 72 hours, or 6 days. IFN-γ, TNF-α and IL-10 were measured using matched antibody pairs (BD Pharmingen, Franklin Lakes, NJ, USA) by ELISA. The limit of detection for these ELISAs was 31 pg/mL. Background levels in non-stimulated control wells were deducted from antigen-stimulated values to determine antigen specific cytokine responses (with negative values recorded as zero). To control for inter-plate and intra-plate variation, a positive-control supernatant (1∶4 and 1∶8 dilution of PHA stimulated Non Endemic Healthy Controls (NEHC) whole blood pooled supernatant) was used in duplicate on each ELISA plate. The mean variability of these duplicate measurements was 2.53% (intra-plate variation). The coefficient of variation between plates (inter-plate variation) was 20.74% for IFN-γ, 10.78% for TNF-α and 18.04% for IL-10.

### Statistical analysis

Because data were generally not normally distributed (as determined using the Kolgomorov-Smirnov test), data are plotted using box and whiskers (Tukey) plots, and statistical differences (P<0.05) between pairs of groups were determined using nonparametric 2-tailed Mann-Whitney tests. Nominal P-values are presented throughout (i.e. without correction for multiple testing). Plots were generated using GraphPad Prism 5 (San Diago, USA), and statistical analyses were performed using GraphPad Prism 5 or SPSS software v18.0.

## Results

### SLA cytokine responses correlate with disease status

**Figure 1:**
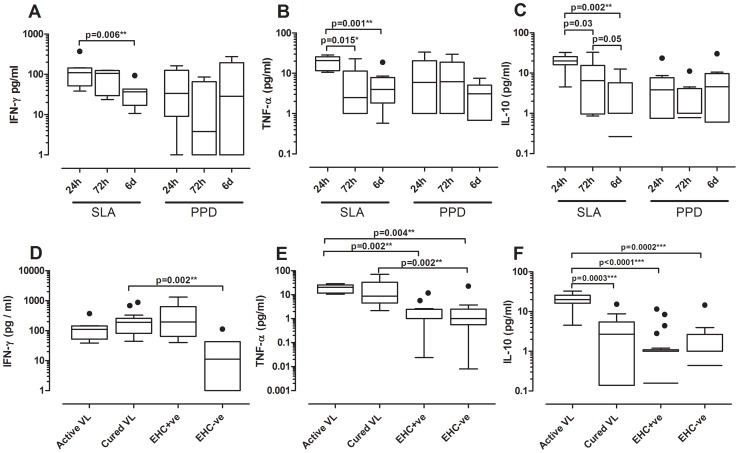
SLA-specific IFN-γ, TNF-α and IL-10 responses. Box plots (Tukey) for cytokine production by peripheral whole blood cells in response to SLA (an Indian *L. donovani* strain, 10 µg/mL), or PPD (5 µg/mL) examined in active VL (n = 8), cured VL (n = 16), EHC^+ve^ (n = 20) and EHC^−ve^ (n = 9) groups by ELISA. (A–C) provide comparisons of cytokine levels over 24 hours, 72 hours and 6 days post stimulation with SLA or PPD in the active VL group (parallel data for IFN-γ responses in the cured VL group are provided in Fig. S1). (D–F) compare cytokine responses at 24 hours post stimulation across the 4 study groups. Antigen-stimulated cytokine responses are provided after subtraction of the non-stimulated control wells. Statistical differences between groups determined using the non-parametric Man-Whitney test are indicated by bars above columns, * indicates p<0.05, ** p<0.01, and *** p<0.001.

To investigate antigen specific production of IFN-γ, TNF-α and IL-10 cytokines, diluted whole blood from different patient groups was initially stimulated with SLA from an Indian *L. donovani* strain. Comparison of responses over time post stimulation in active VL cases showed that all 3 cytokines were highest, and less variable, at the 24 hour time point (Fig. 1A–C). Active cases made variable responses to PPD reflecting prior exposure to *Mycobacterium* and indicating that ability to make a response to mycobacterial antigens is not compromised in active VL patients. Cured cases made similarly variable responses to PPD (Fig. S1). Between group comparisons at 24 hours post stimulation showed that active VL, cured VL, and EHC^+ve^ study groups all made higher IFN-γ responses relative to EHC^−ve^ subjects (Fig. 1D). The observation that active VL cases make a significant amount of IFN-γ is in line with our recent observations for whole blood assays using undiluted blood in a modified Quantiferon assay [6], [23]. Of interest, while cured VL and active VL groups generated TNF-α concomitant with IFN-γ, the EHC^+ve^ group did not (Fig. 1E), suggesting that production of this cytokine might relate to the pathogenic role of TNF-α in VL disease [29]. Note, however, that this was not true for responses to putative vaccine candidates outlined below, which all elicited TNF-α concomitant with IFN-γ in the EHC^+ve^ group. Importantly, only the active VL group made IL-10 in response to Indian *L. donovani* SLA (Fig. 1F), supporting previous data indicating that IL-10 is a key regulatory cytokine in VL patients [10], [30].

**Figure 2:**
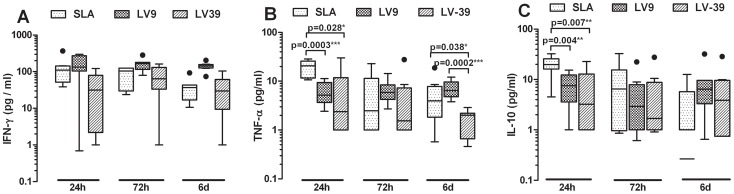
Cross-species cytokine responses to crude leishmania antigens. Box plots (Tukey) for (A) IFN-γ, (B) TNF-α and (C) IL-10 responses in peripheral whole blood cells from active VL patients (n = 8) in response to SLA (an Indian *L. donovani* strain, 10 µg/mL), LV9 (Ethiopian *L. donovani* strain, 10 µg/mL), LV39 (*L. major* strain, 10 µg/mL). Antigen-stimulated cytokine responses are provided after subtraction of the non-stimulated control wells. Statistical differences between groups determined using the non-parametric Man-Whitney test are indicated by bars above columns, * indicates p<0.05, ** p<0.01, and *** p<0.001.

Cytokine responses to 3 different *Leishmania* strains, an Indian *L. donovani* strain (designated SLA), an Ethiopian *L. donovani* strain (LV9) and *L. major* strain (LV39) were compared (Fig. 2). For IFN-γ responses the two *L. donovani* preparations stimulated equivalent responses (Fig. 2A). Interestingly, SLA prepared from the local Indian *L. donovani* strain elicited significantly stronger 24 h TNF-α (Fig. 2B) and IL-10 responses (Fig. 2C) compared to Ethiopian *L. donovani* (p = 0.0003; p = 0.004) or the *L. major* strain (p = 0.028; p = 0.007). The *L. major* antigen was more variable in eliciting responses across all cytokines and time points.

The ability of diluted whole blood assay samples to respond to mitogenic stimulation with PHA (Tables S1 and S2) confirmed the viability of the cells from all donors.

### Cytokine responses to TRYP and LACK recombinant proteins

We previously demonstrated that high CD4-derived IFN-γ to low IL-10 ratios predicted vaccine success in mice when comparing TRYP and LACK as DNA with/without Modified Vaccinia Ankara vaccines [12], [31]. Here, we examined immune responses to these potential vaccine antigens in clinically well characterized groups of human subjects. The full set of results for IFN-γ, TNF-α and IL-10 responses to TRYP (Fig. S2) and LACK (Fig. S3) at 24 hours, 72 hours and 6 days post stimulation in active VL, cured VL, EHC^+ve^ and EHC^−ve^ study groups is provided in the figures S2 and S3. Of note, although the EHC^−ve^ group comprised negative responders to Indian SLA by the modified Quantiferon assay, their cytokine responses to TRYP and LACK was rarely significantly different as a group from cured VL and EHC^+ve^ groups. Hence, we conclude that there are exposed individuals amongst this EHC group, although we did not test non-endemic healthy control responses to these antigens. As this exposure status is variable and equivocal, we exclude them from further analysis of between group responses.

**Figure 3:**
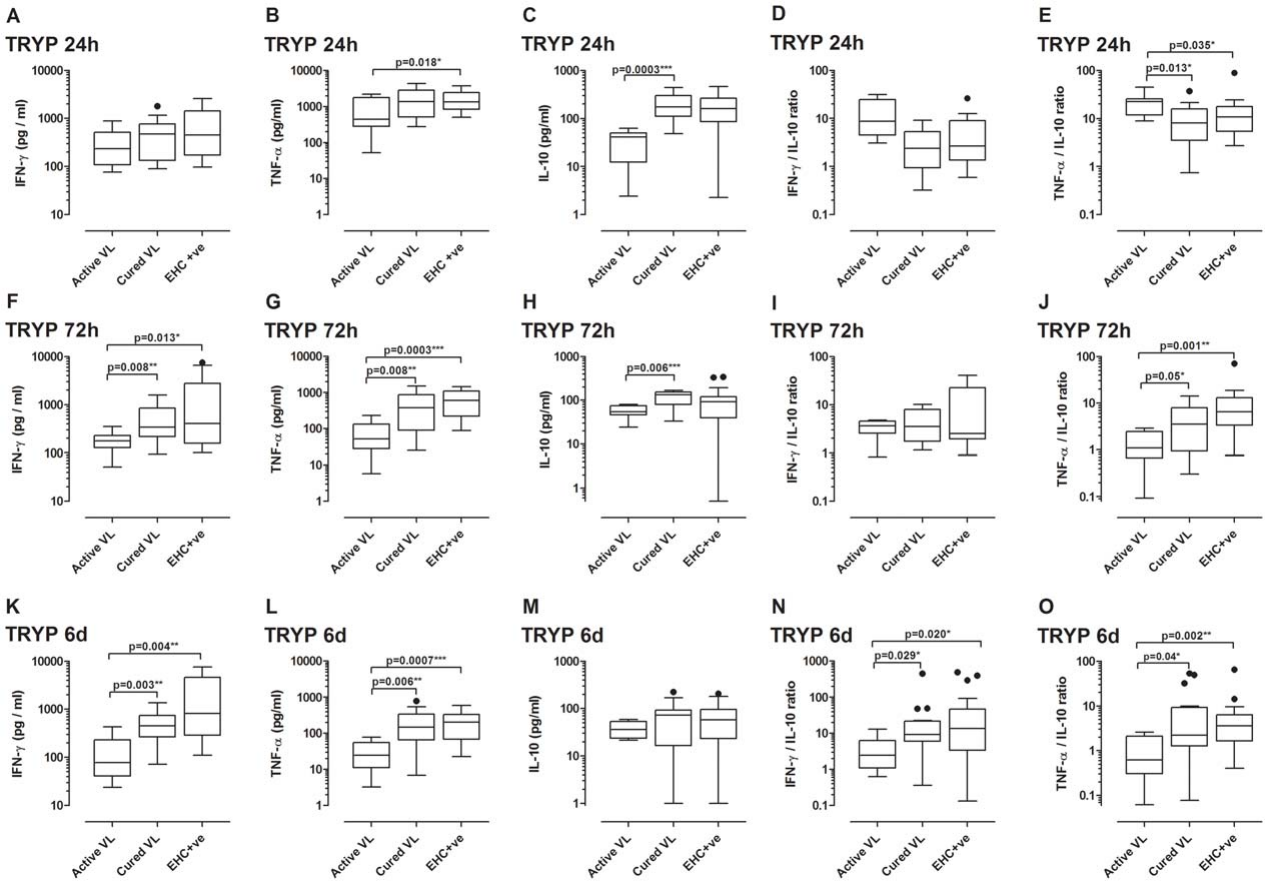
TRYP-specific cytokine release in whole blood assays. Box plots (Tukey) for TRYP recombinant protein (10 µg/mL) stimulated cytokine release at (A–E) 24 hours, (F–G) 72 hours, and (K–O) 6 days post stimulation in active VL (n = 8), cured VL (n = 20) and EHC^+ve^ (n = 20) study groups (full data for all study groups are provided in Fig. S2). Data are presented for each cytokine response at the different time points (A,F,K IFN-γ; B,G,L TNF-α; C,H,M IL-10) as well as for the ratios of IFN-γ to IL-10 (D,I,N) and TNF-α to IL-10 (E,J,O). Antigen-stimulated cytokine responses are provided after subtraction of the non-stimulated control wells. Statistical differences between groups determined using the non-parametric Man-Whitney test are indicated by bars above columns, * indicates p<0.05, ** p<0.01, and *** p<0.001.

Results for active VL, cured VL and EHC^+ve^ groups are summarised in figures 3 (TRYP) and 4 (LACK). For both antigens, the pattern of IFN-γ and TNF-α responses across the 3 groups is generally established at 24 hours, and clear cut by 72 hours and 6 days, post stimulation. For these two cytokines, responses were significantly lower in active VL compared to cured VL and EHC^+ve^ groups at 72 hours and 6 days post stimulation. The pattern of responses for IL-10 was similar (i.e. higher in cured VL and EHC^+ve^ compared to active VL), but more clearly apparent at 24 hours post-stimulation. This led to interesting between group differences in the ratios of IFN-γ to IL-10 and TNF-α to IL-10 at 24 hours, when ratios were significantly higher in the active VL group compared to cured VL and EHC^+ve^ groups (particularly for TRYP), compared to 6 days of stimulation where the reverse was true for both antigens. For both antigens, the ratios of IFN-γ to IL-10 and TNF-α to IL-10 were highest in the EHC^+ve^ group, suggesting that a potent pro-inflammatory response relative to modest levels of IL-10 may correlate with protection from disease in this confirmed Quantiferon positive exposed EHC group.

**Figure 4:**
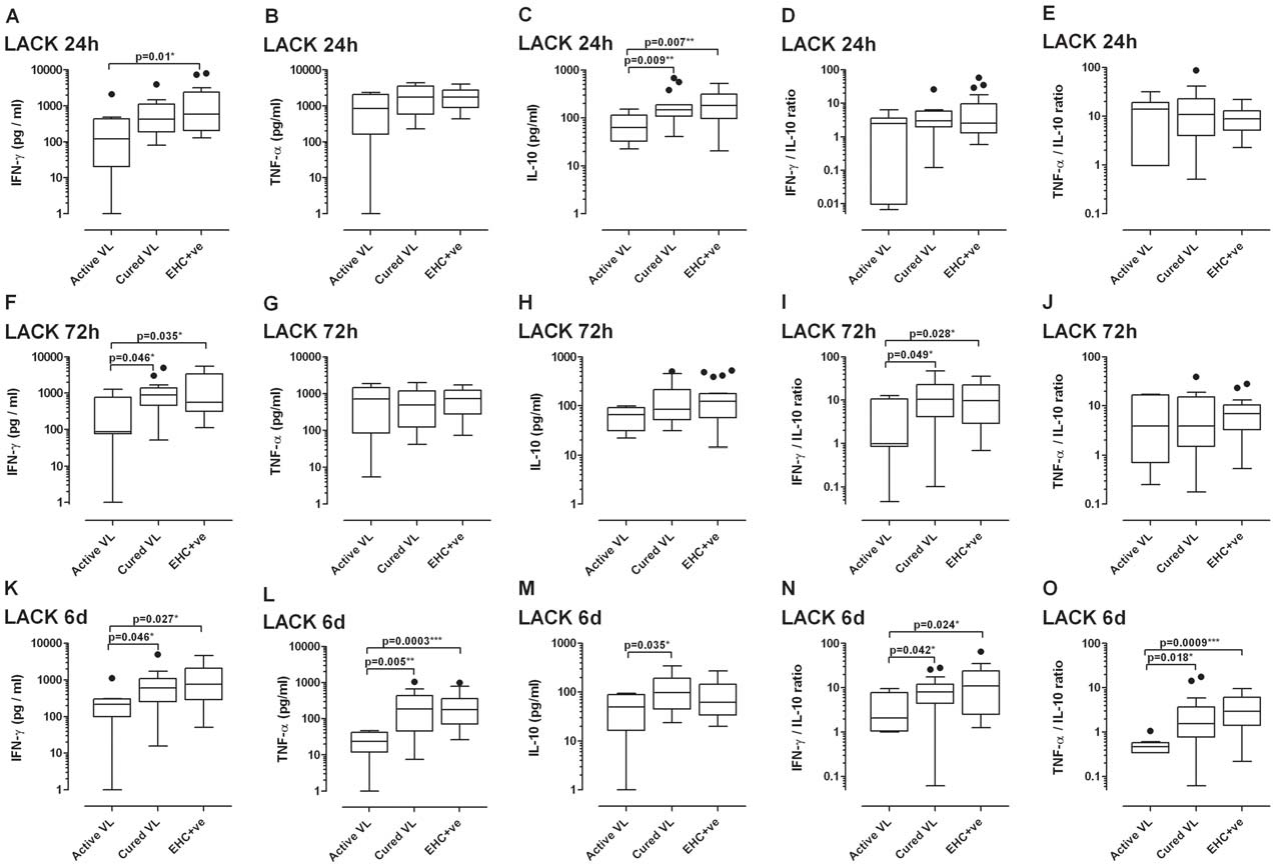
LACK-specific cytokine release in whole blood assays. Box plots (Tukey) for LACK recombinant protein (10 µg/mL) stimulated cytokine release at (A–E) 24 hours, (F–G) 72 hours, and (K–O) 6 days post stimulation in active VL (n = 8), cured VL (n = 20) and EHC^+ve^ (n = 20) study groups (full data for all study groups are provided in Fig. S3). Data are presented for each cytokine response at the different time points (A,F,K IFN-γ; B,G,L TNF-α; C,H,M IL-10) as well as for the ratios of IFN-γ to IL-10 (D,I,N) and TNF-α to IL-10 (E,J,O). Antigen-stimulated cytokine responses are provided after subtraction of the non-stimulated control wells. Statistical differences between groups determined using the non-parametric Man-Whitney test are indicated by bars above columns, * indicates p<0.05, ** p<0.01, and *** p<0.001.

### Antigen specific cytokine release in response to novel vaccine antigens

**Figure 5:**
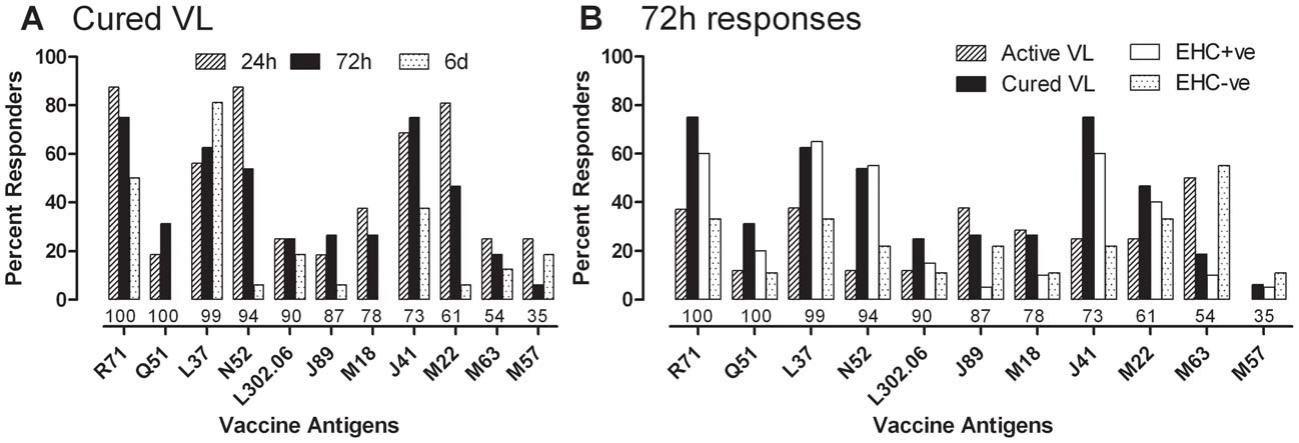
IFN-γ responses to peptide pools for 11 novel defined leishmania antigens. Bar graphs indicating the percentage of responders to peptide pools (5 µg/mL) for each of 11 novel antigens (A) at 24 hours, 72 hours and 6 days post stimulation for the cured VL group, and (B) across active VL (n = 8), cured VL (n = 16), EHC^+ve^ (n = 20) and EHC^−ve^ (n = 9) groups at the 72 hour time point. Numbers immediately under the bars indicate the percent identity between the originally sequenced *L. major* proteins and their *L. infantum* orthologues. See also tables S1 and S2.

**Figure 6:**
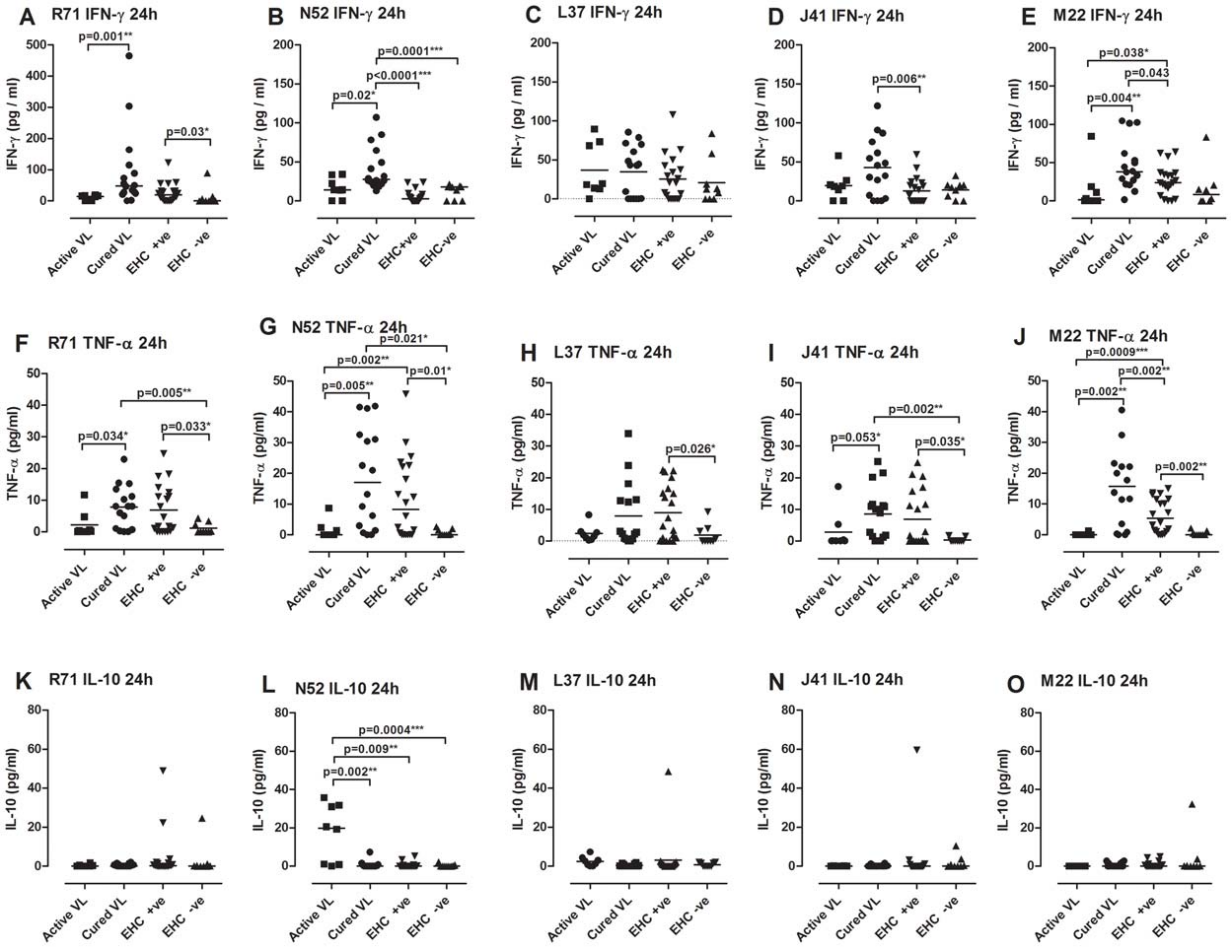
IFN-γ, TNF-α and IL-10 responses to peptide pools for 5 novel antigens. Dot plots showing individual cytokine responses in subjects from active VL (n = 8), cured VL (n = 16), EHC^+ve^ (n = 20) and EHC^−ve^ (n = 9) groups 24 hours post stimulation of whole blood assays with peptide pools (5 µg/mL) for the 5 antigens R71, L37, N52, J41, and M22. Data are presented for each cytokine response (A–E IFN-γ; F–J TNF-α; K–O IL-10). Bars indicate the mean group response. Antigen-stimulated cytokine responses are provided after subtraction of the non-stimulated control wells. Statistical differences between groups determined using the non-parametric Man-Whitney test are indicated by bars above columns, * indicates p<0.05, ** p<0.01, and *** p<0.001. Data for 72 hours and 6 days post stimulation are presented in Fig. S4 and Fig. S5, respectively.

We previously identified 14 protective *Leishmania* antigens in a screen of 100 candidates delivered as DNA vaccines to susceptible BALB/c mice [25]. We measured IFN-γ and TNF-α as effector pro-inflammatory cytokine responses to peptide pools for each of 11 of these antigens in diluted whole blood assays, and IL-10 as a measure of their ability to elicit a regulatory cytokine response. A full summary of responder status on a categorical scale (− = <20 pg/ml; + = 20–99 pg/ml; ++ = 100–249; +++ = 250–499 pg/ml; ++++ = 500–10000 pg/ml; ++++ = >10000 pg/ml) to each of the 11 antigens, and to control SLA, PPD and PHA stimulations, is provided for all individuals in Tables S1 and S2. As these antigens were based on *L. major* sequence data, the antigens are presented throughout in order of their percent identity to *L. infantum*, as reported by us previously [28]. As would be predicted on the basis of genetic heterogeneity in HLA-restricted T cell responses and other background genetic and environmental factors, not all individuals make a response to individual candidate vaccine antigens. Using cured VL patients as an initial evaluation of percent responders (≥20 ng/mL above background) with time post stimulation, we observed maximal IFN-γ responders at 24 hours and 72 hours post stimulation (Fig. 5A), with 55–87.5% responders to 5 of the novel antigens (R71, L37, N52, J41 and M22; of these L37 exceptional in eliciting the highest sustained IFN-γ responses at day 6 post stimulation, see also Table S2). Comparing across groups for the 72 hour time point (Fig. 5B), we observed 40–65% responders to these 5 novel antigens in the EHC^+ve^ group, with ≥25% of active VL cases also making IFN-γ responses to these antigens. Looking across cytokine responses for these 5 antigens (Fig. 6), we observe a similar profile of TNF-α responses in cured VL and EHC^+ve^ groups as we observed for IFN-γ, but no IL-10. Even amongst active VL cases, N52 was the only antigen to elicit IL-10 responses (Fig. 6L). As for TRYP and LACK, a small number (22–33%) of responders was observed in the EHC^−ve^ group, consistent with evidence of exposure in these individuals despite their negative response in the modified Quantiferon assay. Alternatively, these might represent non-specific responses to these antigens as we did not include non-endemic controls in our study. In summary, we have identified five *Leishmania* antigens from 11 putative vaccine candidates tested that stimulate potent pro-inflammatory recall responses in exposed but protected individuals (cured VL patients and EHC^+ve^) in the absence of regulatory IL-10, providing potential immunotherapeutic or vaccine targets for future investigation.

## Discussion

A variety of defined antigens have been investigated as vaccine antigen candidates for VL in animal models [32], [33], [34], but few have advanced to human clinical trials [35], [36]. One limitation in the search for an effective vaccine for leishmaniasis is the lack of information on immunological correlates of natural and vaccine-mediated protection in humans. In recent studies we have highlighted the use of a modified Quantiferon assay to screen for naturally exposed resistant individuals in the Indian study area [6]. That assay relies on 3 mL of undiluted whole blood. Here we show that individuals positive by the modified Quantiferon assay are also positive in our 96-well plate assays using diluted whole blood, providing the means to more efficient screening in large-scale epidemiological studies as has been used previously in studies of mycobacterial diseases [37], [38], [39]. Importantly too, our 96-well plate assay also showed that active VL patients were positive for IFN-γ in these diluted whole blood 96-well plate assays. Our initial demonstration [23] that active VL patients are positive for IFN-γ in the modified Quantiferon assay was remarkable given the numerous previous studies that had failed to observe cellular proliferation or IFN-γ release after stimulation of peripheral blood mononuclear cells from active VL patients with crude *Leishmania* antigen [10], [40], [41], [42]. Ability to measure this IFN-γ response in the diluted whole blood assay described here will also facilitate more efficient screening of active VL cases using smaller blood volumes in a 96-well plate format.

In human and murine cells infected *in vitro*, and in mice *in vivo*, clearance of *Leishmania* parasites requires IFN-γ. However, IFN-γ alone does not predict vaccine-mediated protection in mice [12], [31], [43], [44]. Rather, the simultaneous production of IFN-γ, IL-2 and TNF-α by a particular subset of CD4 T cells [11], and/or the balance between pro-inflammatory IFN-γ/TNF-α and regulatory IL-10 [12], [31], [44], [45], have been variously shown to be predictive of vaccine outcome. Epidemiological studies indicate that patients drug-cured from *L. donovani* infection are protected against subsequent clinical disease [46], and it is thought that exposed individuals who test as positive to crude leishmanial antigens in the modified Quantiferon assay employed in our study area in India are infected asymptomatic individuals who are resistant to developing active VL disease [6]. Therefore, in the analysis of human immune responses to known and novel antigens presented here, we hypothesized that ability to stimulate IFN-γ, TNF-α and IL-10 in cured VL and EHC^+ve^ individuals, compared to active VL cases, would provide some insight into their potential as vaccine candidates.

Our investigations focused initially on the known vaccine candidates TRYP and LACK. Although others have found LACK protective in murine models of cutaneous leishmaniasis [47], in the virulent model of visceralising *L. major* LV39 infection in mice we found that TRYP was protective but LACK was not [12]. Although the vaccine-induced IFN-γ responses were similar between the two antigens in mice, lower IL-10 was elicited by TRYP than LACK, resulting in higher IFN-γ to IL-10 ratios as correlates of protective immunity. In the human studies described here, we found that TRYP and LACK were equivalent to each other in the magnitudes of IFN-γ, TNF-α and IL-10 responses elicited, and in generating higher IFN-γ to IL-10 and TNF-α to IL-10 ratios in putatively protected cured VL and EHC^+ve^ individuals than in active VL cases. It was of interest that in India, the asymptomatic EHC^+ve^ group had equivalent responses to the cured VL group, whereas in our recent study [28] of the same antigens (and antigen preparations) in Brazil, we found that asymptomatic DTH^+ve^ individuals had lower ratios of IFN-γ to IL-10 and TNF-α to IL-10 compared to cured VL patients. This was due to higher IL-10 responses in the DTH^+ve^ group compared to the cured VL group, leading us to suggest that a measure of modulation of the pro-inflammatory response by IL-10 in the DTH^+ve^ group might contribute to the protective response. In active VL disease, high levels of TNF-α contribute to fever and cachexia, and are detrimental [29], and it is not yet known what role in pathogenesis is played by the strong 24 hour IFN-γ responses observed in whole blood assays in active VL [6], [23].

In our analysis of novel vaccine candidates, we found that 5 antigens (R71, L37, N52, J41 and M22) elicited IFN-γ and TNF-α responses in a high percentage of cured VL (55–87.5%) and EHC^+ve^ (40–65%) subjects. This represents remarkable replication of recent findings from an area endemic for *L. infantum chagasi* in northern Brazil, where 4 of these antigens (R71, L37, N52 and M22; same preparations of peptide pools) also elicited strong IFN-γ and TNF-α responses in both cured VL and exposed asympotmatic DTH+ individuals [28]. In Brazil, responses to J41 were only observed in the cured VL group, but the sample size for DTH+ individuals was small (n = 4). Strong responses were also observed in Brazil to two additional antigens, L302.06 and M18, for which a lower percentage (<30%) of responders were observed in India. This may reflect small samples sizes, differences in amino acid sequences of the parasites, and/or differences in HLA alleles between the two populations. On balance, all of these antigens remain strong candidates in the context of a multivalent cross-species vaccine against leishmaniasis. R71 and L37 are ribosomal proteins with high (100 and 99%, respectively) percentage identity at the amino acid level between *L. major* and *L. infantum* [28]. N52 is a V-ATPase subunit F which also has high (94%) identity across the two species. J41 and M22 are hypothetical proteins of unknown function which, despite lower percent identities (73% and 61%, respectively) between *L. major* and *L. infantum*, appear to provide cross-reactive epitopes that are recognised in both Brazil [28] and India. An important contrast between the two endemic regions was the almost complete lack of IL-10 responses to these novel antigens (same preparations of peptide pools) in the Indian study in both cured VL and EHC^+ve^ groups, whereas in the Brazilian study cured VL subjects who were positive for IFN-γ and TNF-α responses also produced IL-10. N52 was also unique in being the only antigen to stimulate IL-10 responses in active VL patients, suggesting that responses to this antigen might provide an important early diagnostic biomarker for disease-associated IL-10 in VL. Further studies are needed to evaluate more carefully the differences in cytokine responses to individual antigens in active compared to cured VL groups, as well as between cured VL and exposed asymptomatic DTH+ or modified Quantiferon positive groups. Unlike Brazil [4], [5], DTH responses have not provided a sensitive means of evaluating cell mediated immune response in cured VL or exposed individuals in India [48], pointing to potential differences in cell-mediated responses between DTH+ compared to Quantiferon positive exposed asymptomatic individuals that might hold the key to uncovering the true correlates of vaccine-induced immunity in leishmaniasis.

Results of our study demonstrate that only a percentage of individuals respond to vaccine antigens that have individually been shown to be protective in mice. This suggests that defined vaccine for use in humans will need to be complex multi-epitope/antigens vaccines. To date, only one multicomponent vaccine, Leish-111f, has been assessed in a large clinical trial [49]. Our recent small-scale clinical trial in a *L. donovani* endemic area showed Leish-F1-MPL-SE was safe and well tolerated in people with and without prior VL exposure and induced strong antigen-specific T cell responses [36]. The data presented here, and in our earlier study from Brazil [28], provide evidence to support a number of novel candidates that could be taken forward as vaccines against human leishmaniasis.

## Supporting Information

Figure S1 **Box plots (Tukey) for IFN-γ production by peripheral whole blood cells in response to SLA (an Indian** ***L. donovani*** **strain, 10 µg/mL), or PPD (5 µg/mL) as measured by ELISA.** The IFNγ responses in (A) the active VL group (n = 8) are compared with (B) the cured VL group (n = 16), over 24 hours, 72 hours and 6 days post stimulation with SLA or PPD. The data presented in main figure 1 are a subset of the data presented here. Statistical differences between groups determined using the non-parametric Man-Whitney test are indicated by bars above columns, * indicates p<0.05, ** p<0.01, and *** p<0.001.
(PDF)

Figure S2 **Box plots (Tukey) for TRYP recombinant protein (10 µg/mL) stimulated cytokine release at (A–E) 24 hours, (F–G) 72 hours, and (K–O) 6 days post stimulation in active VL (n = 8), cured VL (n = 20), EHC^+ve^ (n = 20) and EHC-ve (n = 9) study groups.** Data are presented for each cytokine response at the different time points (A,F,K IFN-γ; B,G,L TNF-α; C,H,M IL-10) as well as for the ratios of IFN-γ to IL-10 (D,I,N) and TNF-α to IL-10 (E,J,O). Statistical differences between groups determined using the non-parametric Man-Whitney test are indicated by bars above columns, * indicates p<0.05, ** p<0.01, and *** p<0.001.
(PDF)

Figure S3 **Box plots (Tukey) for LACK recombinant protein (10 µg/mL) stimulated cytokine release at (A–E) 24 hours, (F–G) 72 hours, and (K–O) 6 days post stimulation in active VL (n = 8), cured VL (n = 20), EHC^+ve^ (n = 20) and EHC-ve (n = 9) study groups.** Data are presented for each cytokine response at the different time points (A,F,K IFN-γ; B,G,L TNF-α; C,H,M IL-10) as well as for the ratios of IFN-γ to IL-10 (D,I,N) and TNF-α to IL-10 (E,J,O). Statistical differences between groups determined using the non-parametric Man-Whitney test are indicated by bars above columns, * indicates p<0.05, ** p<0.01, and *** p<0.001.
(PDF)

Figure S4 **Dot plots showing individual cytokine responses in subjects from active VL (n = 8), cured VL (n = 16), EHC^+ve^ (n = 20) and EHC^−ve^ (n = 9) groups 72 hours post stimulation of whole blood assays with peptide pools (5 µg/mL) for the 5 antigens R71, L37, N52, J41, and M22.** Data are presented for each cytokine response (A–E IFN-γ; F–J TNF-α; K–O IL-10). Bars indicate the mean group response. Statistical differences between groups determined using the non-parametric Man-Whitney test are indicated by bars above columns, * indicates p<0.05, ** p<0.01, and *** p<0.001.
(PDF)

Figure S5 **Dot plots showing individual cytokine responses in subjects from active VL (n = 8), cured VL (n = 16), EHC^+ve^ (n = 20) and EHC^−ve^ (n = 9) groups 6 days post stimulation of whole blood assays with peptide pools (5 µg/mL) for the 5 antigens R71, L37, N52, J41, and M22.** Data are presented for each cytokine response (A–E IFN-γ; F–J TNF-α; K–O IL-10). Bars indicate the mean group response. Statistical differences between groups determined using the non-parametric Man-Whitney test are indicated by bars above columns, * indicates p<0.05, ** p<0.01, and *** p<0.001.
(PDF)

Table S1 **IFN-γ responses in individuals cured from VL at 24 hours, 72 hours, and 6 days after stimulation of whole blood** ***ex vivo*** **with candidate vaccine antigens (peptide pools; 5 µg/mL), SLA (10 µg/mL), PPD (5 µg/mL), or mitogen PHA (5 µg/mL).** IFN-γ levels were measured by ELISA. Percent identity with *L. infantum* sequence at the amino acid level is indicated below candidate vaccine antigens (R71, Q51, etc.), which were originally derived from *L. major*.
(PDF)

Table S2 **IFN-γ responses in EHC +ve (EC-01 to EC-20), active VL (Vl-1 to VL-8) and EHC-ve (EC-42 to EC-50) individuals at 72 hours after stimulation of whole blood** ***ex vivo*** **with candidate vaccine antigens (peptide pools; 5 µg/mL), SLA (10 µg/mL), PPD (5 µg/mL), or mitogen PHA (5 µg/mL).** IFN-γ levels were measured by ELISA.
(PDF)
