# Supplementary figures and images for: Cytokine Responses to Novel Antigens in an Indian Population Living in an Area Endemic for Visceral Leishmaniasis
Source: PLoS Negl Trop Dis. 2012 Oct 25;6(10):e1874. doi: 10.1371/journal.pntd.0001874 (PMC3493615; doi:10.1371/journal.pntd.0001874)

# Supplementary Figure S1

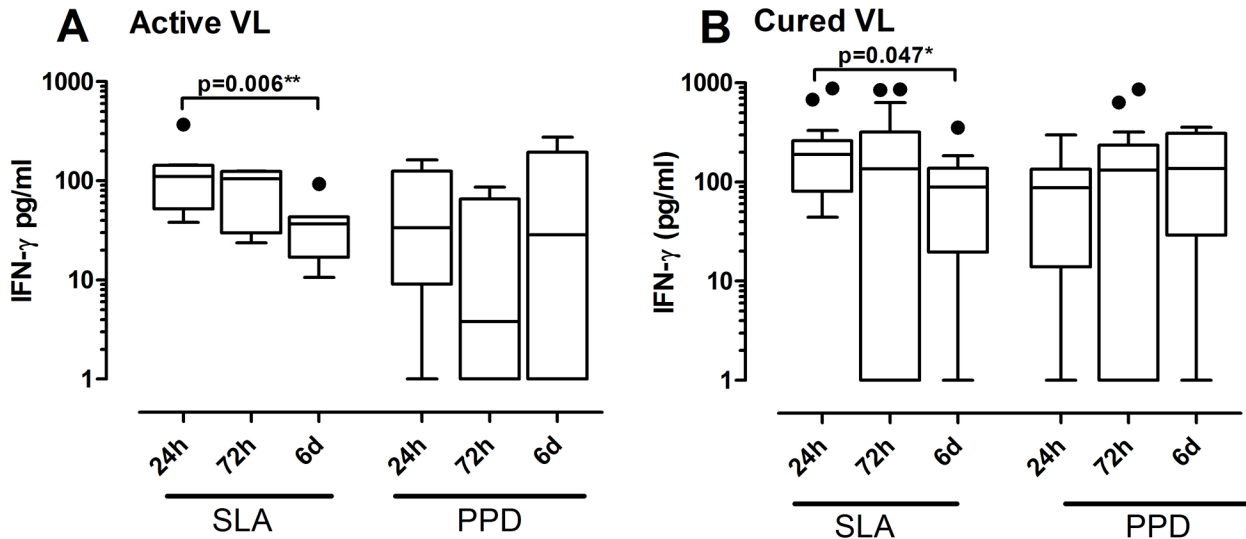

Supplement: Figure S1 — Box plots (Tukey) for IFN-γ production by peripheral whole blood cells in response to SLA (an Indian L. donovani strain, 10 µg/mL), or PPD (5 µg/mL) as measured by ELISA. The IFNγ responses in (A) the active VL group (n = 8) are compared with (B) the cured VL group (n = 16), over 24 hours, 72 hours and 6 days post stimulation with SLA or PPD. The data presented in main figure 1 are a subset of the data presented here. Statistical differences between groups determined using the non-parametric Man-Whitney test are indicated by bars above columns, * indicates p<0.05, ** p<0.01, and *** p<0.001. (PDF) [file pntd.0001874.s001.pdf]

# Supplementary Figure S2

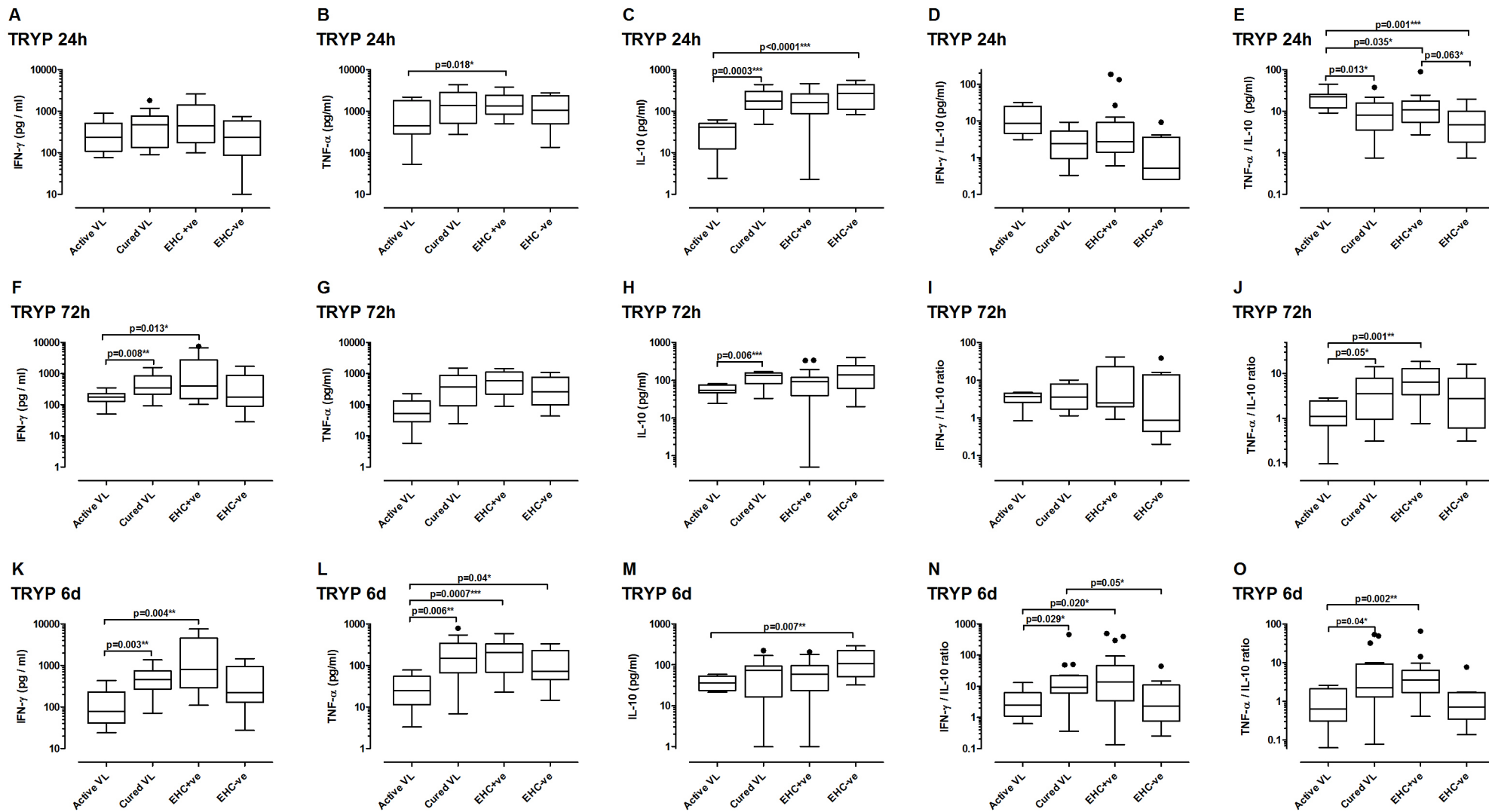

Supplement: Figure S2 — Box plots (Tukey) for TRYP recombinant protein (10 µg/mL) stimulated cytokine release at (A–E) 24 hours, (F–G) 72 hours, and (K–O) 6 days post stimulation in active VL (n = 8), cured VL (n = 20), EHC+ve (n = 20) and EHC-ve (n = 9) study groups. Data are presented for each cytokine response at the different time points (A,F,K IFN-γ; B,G,L TNF-α; C,H,M IL-10) as well as for the ratios of IFN-γ to IL-10 (D,I,N) and TNF-α to IL-10 (E,J,O). Statistical differences between groups determined using the non-parametric Man-Whitney test are indicated by bars above columns, * indicates p<0.05, ** p<0.01, and *** p<0.001. (PDF) [file pntd.0001874.s002.pdf]

# Supplementary Figure S3

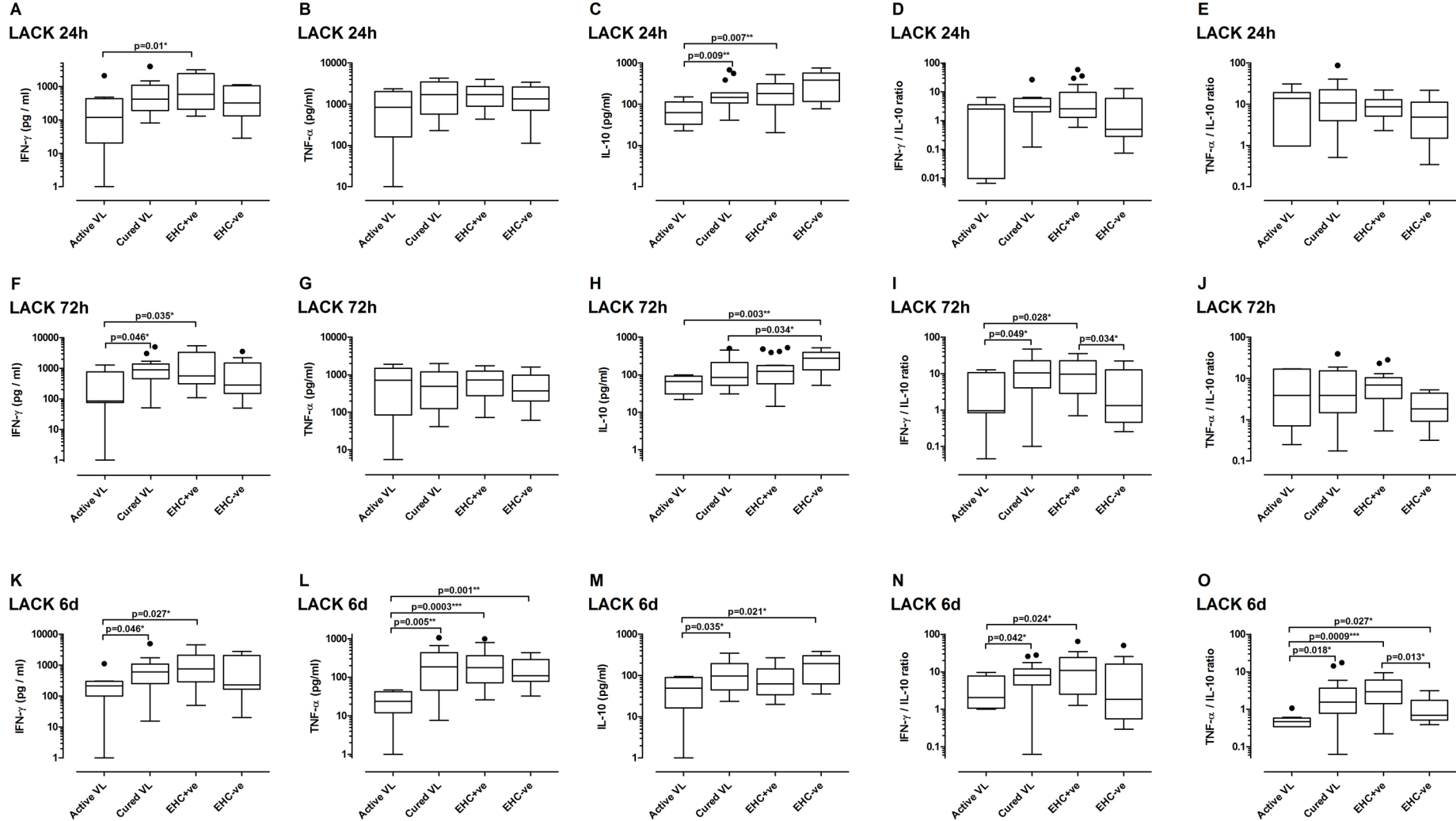

Supplement: Figure S3 — Box plots (Tukey) for LACK recombinant protein (10 µg/mL) stimulated cytokine release at (A–E) 24 hours, (F–G) 72 hours, and (K–O) 6 days post stimulation in active VL (n = 8), cured VL (n = 20), EHC+ve (n = 20) and EHC-ve (n = 9) study groups. Data are presented for each cytokine response at the different time points (A,F,K IFN-γ; B,G,L TNF-α; C,H,M IL-10) as well as for the ratios of IFN-γ to IL-10 (D,I,N) and TNF-α to IL-10 (E,J,O). Statistical differences between groups determined using the non-parametric Man-Whitney test are indicated by bars above columns, * indicates p<0.05, ** p<0.01, and *** p<0.001. (PDF) [file pntd.0001874.s003.pdf]

# Supplementary Figure S4

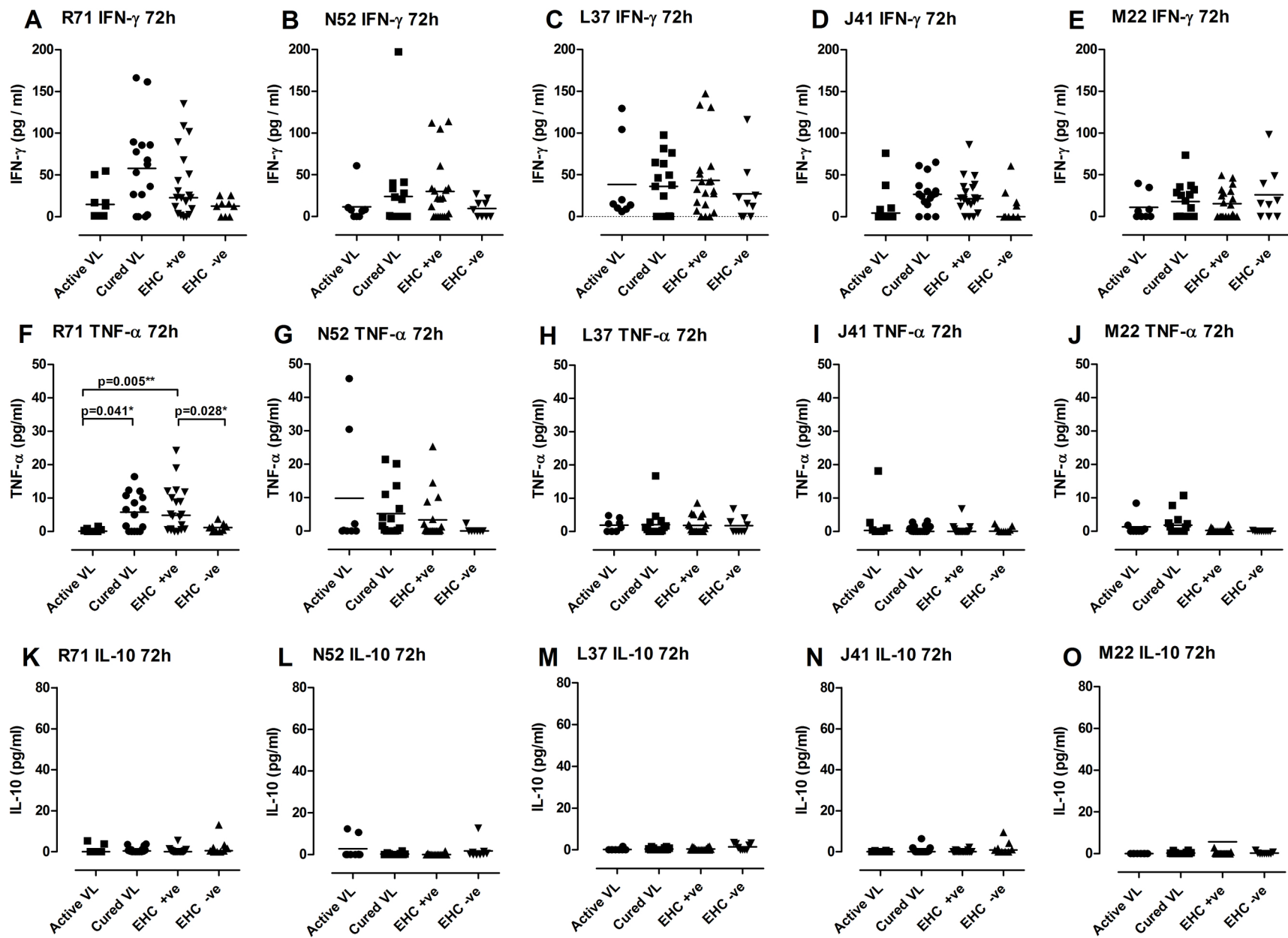

Supplement: Figure S4 — Dot plots showing individual cytokine responses in subjects from active VL (n = 8), cured VL (n = 16), EHC+ve (n = 20) and EHC−ve (n = 9) groups 72 hours post stimulation of whole blood assays with peptide pools (5 µg/mL) for the 5 antigens R71, L37, N52, J41, and M22. Data are presented for each cytokine response (A–E IFN-γ; F–J TNF-α; K–O IL-10). Bars indicate the mean group response. Statistical differences between groups determined using the non-parametric Man-Whitney test are indicated by bars above columns, * indicates p<0.05, ** p<0.01, and *** p<0.001. (PDF) [file pntd.0001874.s004.pdf]

# Supplementary Figure S5

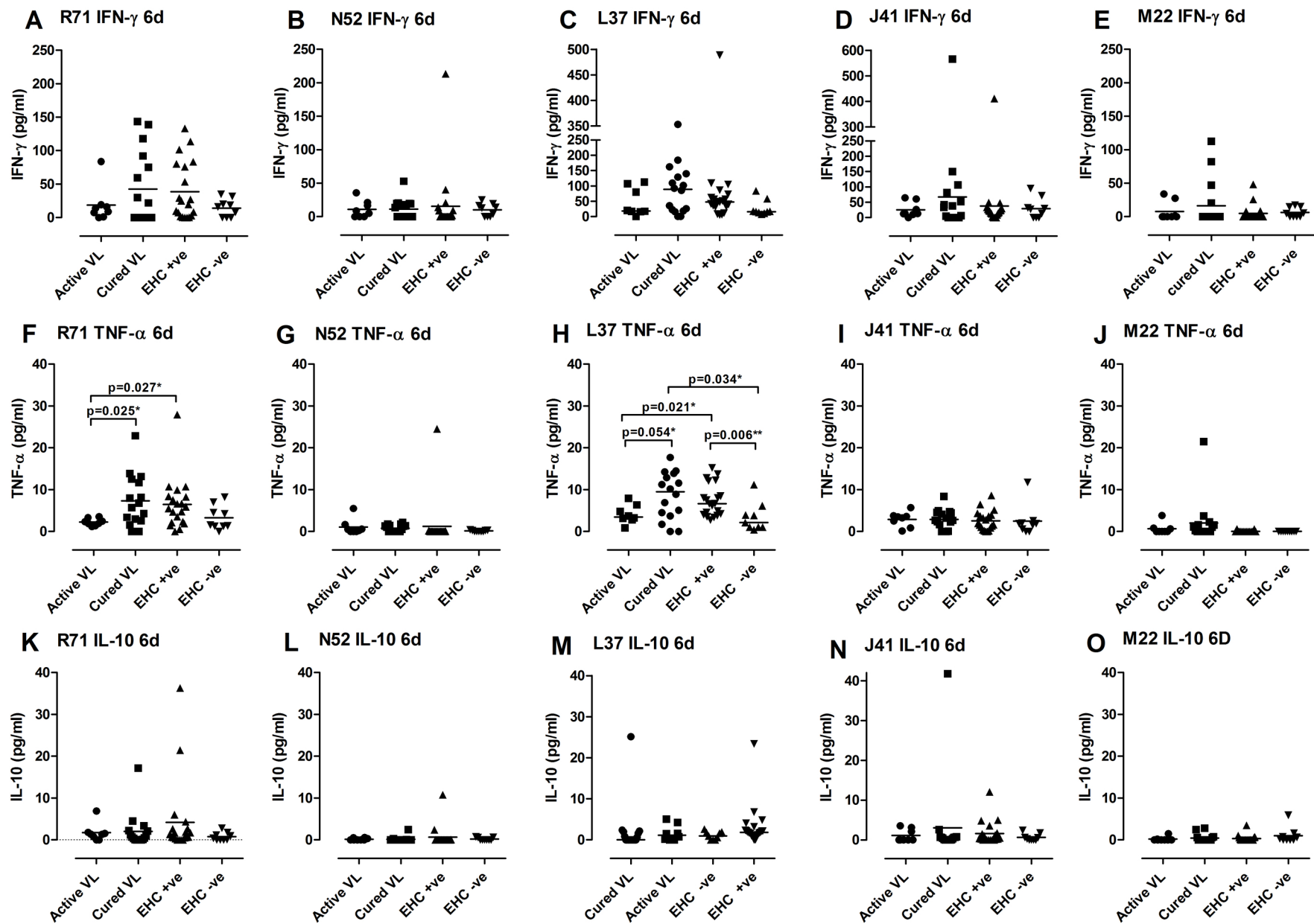

Supplement: Figure S5 — Dot plots showing individual cytokine responses in subjects from active VL (n = 8), cured VL (n = 16), EHC+ve (n = 20) and EHC−ve (n = 9) groups 6 days post stimulation of whole blood assays with peptide pools (5 µg/mL) for the 5 antigens R71, L37, N52, J41, and M22. Data are presented for each cytokine response (A–E IFN-γ; F–J TNF-α; K–O IL-10). Bars indicate the mean group response. Statistical differences between groups determined using the non-parametric Man-Whitney test are indicated by bars above columns, * indicates p<0.05, ** p<0.01, and *** p<0.001. (PDF) [file pntd.0001874.s005.pdf]
