# Supplementary material for: Cytokine Responses to Novel Antigens in an Indian Population Living in an Area Endemic for Visceral Leishmaniasis
Source: PLoS Negl Trop Dis. 2012 Oct 25;6(10):e1874. doi: 10.1371/journal.pntd.0001874 (PMC3493615; doi:10.1371/journal.pntd.0001874)
Supplement: Table S1 — IFN-γ responses in individuals cured from VL at 24 hours, 72 hours, and 6 days after stimulation of whole blood ex vivo with candidate vaccine antigens (peptide pools; 5 µg/mL), SLA (10 µg/mL), PPD (5 µg/mL), or mitogen PHA (5 µg/mL). IFN-γ levels were measured by ELISA. Percent identity with L. infantum sequence at the amino acid level is indicated below candidate vaccine antigens (R71, Q51, etc.), which were originally derived from L. major. (PDF) [file pntd.0001874.s006.pdf]

Table S1. IFN-γ responses in individuals cured from VL at 24 hours, 72 hours, and 6 days after stimulation of whole blood *ex vivo* with candidate vaccine antigens (peptide pools; 5 µg/mL), SLA (10 µg/mL), PPD (5 µg/mL), or mitogen PHA (5 µg/mL). IFN-γ levels were measured by ELISA. Percent identity with *L. infantum* sequence at the amino acid level is indicated below candidate vaccine antigens (R71, Q51, etc.), which were originally derived from *L. major* [1].

	**Mitogen or *Leishmania* Antigen**																																			
S.N.	**PHA**	**PPD**	**SLA**	**R71 100%**			**Q 51 100%**			**L37 99%**			**N52 94%**			**L302.06 90%**			**J89 87%**			**M18 78%**			**J41 73%**			**M22 61%**			**M63 54%**			**M 57 35%**		
				24 hrs	72 hrs	Day-6	24 hrs	72 hrs	Day-6	24 hrs	72 hrs	Day-6	24 hrs	72 hrs	Day-6	24 hrs	72 hrs	Day-6	24 hrs	72 hrs	Day-6	24 hrs	72 hrs	Day-6	24 hrs	72 hrs	Day-6	24 hrs	72 hrs	Day-6	24 hrs	72 hrs	Day-6	24 hrs	72 hrs	Day-6
**PT-1**	++++	-	+	-	-	-	-	-	-	-	-	-	+	+	+	+	-		-		+	+	-	-	+	+	-	-	-	-	+	-	-	-	-	-
**PT-2**	+++++	+++	+++	+	-	++	-	-	-	+	-	+	+	-	-	++	+	-	-	-	-	+	-	-	-	-	-	+	-	-	-	-	-	++	+	-
**PT-3**	+++++	++	++	+	-	-	+	-	-	+	-	-	+	-	-	-	-	-	+	-	-	+	-	-	+	-	+	+	-	-	+	-	-	-	-	-
**PT-4**	+++++	-	++++	-	-	-	-	-	-	-	-	+	-	-	-	-	-	-	-	-	-	+	-	-	-	-	-	+	-	-	-	-	-	-	-	-
**PT-5**	++++	++++	++	+++	+	-	-	-	-	+	+	-	+	+	-	-	-	-	+	-	-	+	-	-	+	-	-	-	-	-	-	+	+	-	-	-
**PT-6**	++++	-	++	++	+	+	-	+	-	+	+	++	++	+	-	-	-	-	+	+	-	-	-	-	-	+	+	++	+	-	+	+	-	-	-	-
**PT-7**	++++	+	++++	+	++	+	-	-	-	+	+	+	+	+	-	-	-	-	-	+	-	+	+	-	+	+	-	+		-	+	-	-	-	-	-
**PT-8**	+++++	++++	++	++	+	+	-	-	-	+	-	+	+		-	-	-	-	-	-	-	-		-	+	+	+	+	+	-	-	-	-	-	-	-
**PT-9**	+++++	+	++	+	+	++	+	+	-	+	+	+++	+	++	-	-	-	-	-	+	-	-	+	-	-	+	+++	+	+	-	-	+	-	+	-	-
**PT-10**	++++	+	++	+	+	-	-	-	-	-	-	+	+		-	-	+	-	-	+	-	-	-	-	+	+	-	+	-	-	-	-	-	-	-	-
**PT-11**	++++	+	++++	+	+	-	-	-	-	-	+	++	-	-	-	-	-	-	-	-	-	-	-	-	-	+	-	+	+	-	-	-	-	-	-	-
**PT-12**	+++++	+	++++	+++	++	+	-	+	-	-	+	++	++	+	-	-	-	+	-	-	-	-	+	-	+	+	-	++	+	-	-	-	-	-	-	+
**PT-13**	++++	-	+	+	++	-	-	+	-	-	+	+	+		-	-	-	-	-	-	-	-	-	-	+	+	-	-	-	-	-	-	-	-	-	-
**PT-14**	+++++	+	++++	+	+	-	-	-	-	-	+	++	+	+	-	+	+	+	-	-	-	-	+	-	+	+	-	++	+	+	-	-	+	-	-	+
**PT-15**	+++++	+++	+	+	+	++	+	+	-	+	+	++	+	-	-	-	-	-	-	-	-	-	-	-	++	+	++	+	+	-	-	-	-	+	-	-
**PT-16**	++++	+	+++	+	+	+	-	-	-	+	+	++	+	-	-	+	+	+	-	-	-	-	-	-	+	+	+	+	-	-	-	-	-	+	-	+
	100 %	75 %	100 %	87.5%	75.0%	50.0%	18.7%	31.2%	0%	56.2%	62.5%	81.2%	87.5%	53.8%	6.2%	25%	25%	18.7%	18.5%	26.6%	6.2%	37.5%	26.6%	0%	68.7%	75.0%	37.5%	81%	46.6%	6.2%	25%	18.7%	12.5%	25%	6.2%	18.7%

- = <20pg/ml; + = 20-100pg/ml; ++ =100-250; +++ = 250-500pg/ml; ++++ = 500-10000pg/ml; ++++ = >10000pg/ml;
PHA = Phytohaemagglutinin; PPD = purified protein derivative from *Mycobacterium tuberculosis*; SLA = Soluble Leishmania antigen

1. Stober CB, Lange UG, Roberts MT, Gilmartin B, Francis R, et al. (2006) From genome to vaccines for leishmaniasis: screening 100 novel vaccine candidates against murine Leishmania major infection. Vaccine 24: 2602-2616.
