# Supplementary material for: Cytokine Responses to Novel Antigens in an Indian Population Living in an Area Endemic for Visceral Leishmaniasis
Source: PLoS Negl Trop Dis. 2012 Oct 25;6(10):e1874. doi: 10.1371/journal.pntd.0001874 (PMC3493615; doi:10.1371/journal.pntd.0001874)
Supplement: Table S2 — IFN-γ responses in EHC +ve (EC-01 to EC-20), active VL (Vl-1 to VL-8) and EHC-ve (EC-42 to EC-50) individuals at 72 hours after stimulation of whole blood ex vivo with candidate vaccine antigens (peptide pools; 5 µg/mL), SLA (10 µg/mL), PPD (5 µg/mL), or mitogen PHA (5 µg/mL). IFN-γ levels were measured by ELISA. (PDF) [file pntd.0001874.s007.pdf]

Table S2. IFN-γ responses in EHC +ve (EC-01 to EC-20), active VL (Vl-1 to VL-8) and EHC-ve (EC-42 to EC-50) individuals at 72 hours after stimulation of whole blood *ex vivo* with candidate vaccine antigens (peptide pools; 5 µg/mL), SLA (10 µg/mL), PPD (5 µg/mL), or mitogen PHA (5 µg/mL). IFN-γ levels were measured by ELISA.

**Mitogen or *Leishmania* Antigen**												
**ID**	**PHA**	**SLA**	**R71**	**Q51**	**L37**	**N52**	**L302.06**	**J89**	**M18**	**J41**	**M22**	**M63**	**M57**
			100%	100%	99%	94%	90%	87%	78%	73%	61%	54%	35%
EC-01	+++++	+	-	+	+	+	-	-	+	+	-	+	+
EC-02	+++++	++++	-	-	-	-	-	-	+	-	+	-	-
EC-03	+++++	++++	-	-	+	-	-	-	-	-	-	-	-
EC-04	+++++	++++	+	-	+	+	+	+	-	-	-	+	-
EC-05	+++++	+++	-	-	+	-	-	-	-	+	-	-	-
EC-06	+++++	++++	+	-	++	+	-	-	-	+	-	-	-
EC-07	+++++	+	++	-	+	+	-	-	-	+	+	-	-
EC-08	+++++	++++	++	-	-	+	-	-	-	-	-	-	-
EC-09	+++++	+++	+	-	+	+	-	-	-	+	+	-	-
EC-10	+++++	+++	++	-	+	-	-	-	-	+	+	-	-
EC-11	+++++	++++	-	-	-	++	-	-	-	-	-	-	-
EC-12	+++++	++++	++	-	+++	-	-	-	-	+	+	-	-
EC-13	+++++	++++	-	++	-	-	-	-	-	-	-	-	-
EC-14	+++++	++++	+	-	+	+	-	-	-	+	-	-	-
EC-15	+++++	++++	-	-	+	-	-	-	-	+	-	-	-
EC-16	+++++	++++	+	+	++	++	+	-	-	+	+	-	-
EC-17	+++++	++++	-	-	-	+	+	-	-	+	-	-	-
EC-18	+++++	+	+	-	-	-	-	-	-	-	+	-	-
EC-19	+++++	++	+	-	+	++	-	-	-	+	+	-	-
EC-20	+++++	-	+	+	-	-	-	-	-	-	-	-	-
**♦♦**	**100%**	**95 %**	**60%**	**20%**	**65%**	**55%**	**15%**	**5%**	**10%**	**60%**	**40%**	**10%**	**5%**
VL-1	+++++	++	+	+	++	+	-	+	+	+	+	+	-
VL-2	+++++	+	-	-	-	-	-	+		-	-	-	-
VL-3	+++++	++	+	-	++	-	-	+	+	+	+	+	-
VL-4	+++	++	-	-	-	-	-	-	-	-	-	+	-
VL-5	+++++	++	-	-	-	-	-	-	-	-	-	-	-
VL-6	+++++	++	+	-	-	-	++	-	-	-	-	-	
VL-7	+++++	+	-	-	+	-	-	-	-	-	-	+	-
VL-8	+++	+	-	-	-	-	-	-	-	-	-	-	-
**♦♦**	**100%**	**100%**	**37%**	**12%**	**37%**	**12%**	**12%**	**37.5 %**	**28.6 %**	**25%**	**25%**	**50%**	**0%**
EC-42	+++++	-	+	-	+	-	-	-	-	-	-	+	-
EC-43	++++	-	+	-	+	+	+	+	-	+	+	+	-
EC-44	++++	-	-	-	-	+	-	-	+	-	+	+	-
EC-45	++++	-	+	-	+	-	-	-	-	+	+	+	-
EC-46	++	-	-	-	-	-	-	-	-	-	-	-	+
EC-47	++++	-	-	-	-	-	-	+	-	-	-	-	-
EC-48	++++	-	-	-	-	-	-	-	-	-	-	-	-
EC-49	++++	-	-	-	-	-	-	-	-	-	-	-	-
EC-50	+++	-	-	+	-	-	-	-	-	-	-	+	-
**♦♦**	**100%**	**0%**	**33%**	**11%**	**33%**	**22%**	**11%**	**22%**	**11%**	**22%**	**33%**	**55%**	**11%**

- = <20pg/ml; + = 20-100pg/ml; ++ =100-250; +++ = 250-500pg/ml; ++++ = 500-10000pg/ml; ++++ = >10000pg/ml; PHA = Phytohaemagglutinin; PPD = purified protein derivative from *Mycobacterium tuberculosis*; SLA = Soluble Leishmania antigen. ♦♦ indicates percent of responders to each antigen in each study group. Percent identity with *L. infantum* sequence at the amino acid level is indicated in the row below candidate vaccine antigens (R71, Q51, etc.), which were originally derived from *L. major* [1].

1. Stober CB, Lange UG, Roberts MT, Gilmartin B, Francis R, et al. (2006) From genome to vaccines for leishmaniasis: screening 100 novel vaccine candidates against murine Leishmania major infection. Vaccine 24: 2602-2616.
